# AI feedback does not motivate by itself: a motivational-meaning model for university physical education

**DOI:** 10.3389/fpsyg.2026.1951652

**Published:** 2026-10-19

**Authors:** Xiangyu Wang, Yongwei Wang

**Affiliations:** Department of Physical Education, Capital Normal University, Beijing, China

**Keywords:** artificial intelligence, embodied verification, feedback, motor learning, physical education, self-determination theory, self-regulated learning

## Abstract

Artificial intelligence (AI) can convert movement into scores, visual overlays, and corrective prompts, but technical precision does not determine how learners interpret that feedback. We develop a motivational-meaning model for university physical education (PE), integrating self-determination theory, feedback literacy, self-regulated learning, and motor-learning research. Its contribution is a context-specific account of how pedagogical framing, social visibility, and embodied verification organize the interpretation of an AI-feedback event. Informational and controlling meaning are distinguished from subsequent psychological-need experiences. Blocked translation denotes a temporary difficulty establishing a defensible basis for action, rather than a third motivational meaning. The model also proposes self-regulatory appropriation as the transfer of an externally supported feedback process into independent monitoring and adjustment; its distinctiveness from established self-regulation constructs remains an empirical question. Seven proposition groups specify separable predictions concerning framing, actionable information, contestability, evaluative exposure, discrepancy repair, skill transfer, and a secondary pathway to continued participation. The outcome structure distinguishes system engagement, acquisition performance, retention and transfer, affect and well-being, and voluntary physical activity. Continued participation is treated as a distal hypothesis requiring longitudinal evidence and attention to prior activity, health, habit, and opportunity. We outline independent measurement of event interpretation and need experience, tests that separate technical from perceived accuracy, and ethical, multilevel research designs. The model offers testable design principles rather than evidence that AI feedback improves learning or participation.

## 1 Introduction

An AI system can identify a joint angle, compare a movement with a reference, and issue a corrective cue. A learner may treat the cue as evidence for the next attempt, as a demand to satisfy an external evaluator, or as information that conflicts with other evidence and cannot yet guide action. These interpretations need not follow from the output’s numerical precision. Consider the same validated recording and correction presented privately as a tentative suggestion or publicly as a compulsory judgment. The technical content is held constant, whereas its instructional use changes. This contrast motivates the present account of how AI feedback acquires motivational meaning.

The evidence relevant to this problem comes from several settings that should be kept distinct. In education broadly, reviews report potential benefits and substantial variation in AI-assisted feedback, alongside limited transparency about the underlying algorithms (Ba et al., 2025; Wang W. et al., 2026). These findings do not establish effects in university PE. A review specifically addressing digital formative assessment in PE identified 15 eligible studies and only five with retention tests; its scope includes digital approaches beyond AI and educational settings beyond universities (Müller and Wagner, 2026). It supports a concern with performance and self-assessment, while leaving the proposed university-PE mechanism untested. The present article uses such neighboring evidence to develop hypotheses, not to claim that the complete pathway has already been demonstrated in the focal setting.

The broader feedback literature gives reason for caution. Feedback interventions are not invariably beneficial, and their effects depend on where attention is directed and how the feedback is processed (Kluger and DeNisi, 1996). Claims about the benefits of self-controlled practice and enhanced expectancies in motor learning have also been inflated by underpowered designs and reporting bias (McKay et al., 2025). Outside instructional settings, quantifying an activity can increase the amount performed while reducing enjoyment and subsequent engagement because the activity comes to feel more like work (Etkin, 2016). Current and former users of wearable trackers may not differ in exercise behavior even though former users report guilt and doubts about metric accuracy (Nuss and Li, 2021). These findings do not imply that digital feedback is harmful. They show why system use, immediate performance, retained learning, well-being, and continued participation should not be treated as interchangeable outcomes.

University PE makes this theoretical problem particularly visible. Unlike feedback on a private written task, movement feedback concerns a performing body. Competence may be seen by classmates while it is still developing; errors can become socially comparable; and the learner has access to felt movement and task effects that may agree or conflict with the system’s representation. Meta-analytic work on self-determination theory (SDT) in PE emphasizes both the importance of teacher and peer contexts and the distinctive public display of competence in this setting (Vasconcellos et al., 2020). Recent evidence from university PE links perceived ability exposure with evaluation anxiety and lower psychological well-being, although the cross-sectional design permits only associational conclusions (Wang, 2026). When AI makes performance more precisely visible, storable, and shareable, it can intensify either the usefulness or the evaluative consequences of feedback.

Recent examples address augmented-reality feedback in youth sport (He and Wei, 2025), associations involving AI-assisted training among university student-athletes (Zhang and Sun, 2026), and an AI-assisted intervention in adolescent PE (Zhao et al., 2026). Their populations, technologies, and designs differ; none directly tests the complete mechanism proposed here for university PE. We do not claim that the field generally assumes a direct feature-to-outcome relationship or neglects learner interpretation. Established feedback theories already describe how learners judge credibility, compare evidence, manage affect, and decide how to act (Butler and Winne, 1995; Carless and Boud, 2018). The narrower question is how these processes operate when a movement-related output appears authoritative, can circulate beyond the immediate attempt, and may conflict with bodily or task evidence.

We address this question through a motivational-meaning model of AI feedback in university PE. The central claim is that an output has no fixed motivational function independently of its use and interpretation. Pedagogical framing, social visibility, and embodied verification organize the event in which informational or controlling meaning develops. A temporary blockage can occur when a learner cannot establish a defensible basis for the next action. We distinguish these appraisals from subsequent need experiences, regulatory responses, and learning outcomes. Repeated events may support independent monitoring and adjustment, but neither an immediate correction nor a favorable appraisal establishes retention, transfer, or continued physical activity.

The contribution is an integration and context-specific specification of established theories. The model does not introduce the informational–controlling distinction, claim that context-dependent feedback effects are new, or establish new psychological needs. It makes three commitments that can be tested together: interpretation should change when the use of technically matched output changes; verification and a genuine right to challenge output should be separable from technical explanation or feedback choice alone; and independent regulation should be assessed separately from supported performance. The proposed constructs earn a place only if they distinguish observable processes or predictions beyond existing accounts. Seven proposition groups develop these commitments and separate skill-transfer tests from a more tentative continued-participation hypothesis.

## 2 AI feedback in university physical education: from output to event

### 2.1 What counts as AI feedback

We use *AI feedback* to refer to a bounded event in which an AI-enabled system converts movement-related input into evaluative or corrective output that a learner encounters during instruction. The input may be video, kinematic data, wearable-sensor data, or task performance. The output may be a score, classification, movement trace, visual comparison, natural-language explanation, or corrective prompt. The definition includes computer vision, markerless pose estimation, adaptive video analysis, and related systems when their output is presented as evidence about movement quality or how to improve it.

This event-level definition deliberately avoids treating “AI use” as a unitary intervention. Systems differ in validity, latency, specificity, explainability, personalization, and data persistence. The same system can also be embedded differently across lessons: a score may be viewed privately as a tentative cue, projected publicly as a class rank, used by a teacher to open inquiry, or treated as the final basis for grading. These differences are not peripheral implementation details. They constitute the conditions through which a technical output becomes a psychological event.

Our focus is university PE skill learning within teacher–learner interaction. The model is most directly applicable when students are learning or refining an observable movement and AI output enters a cycle of instruction, attempt, interpretation, and reattempt. It does not assume that AI replaces the teacher. On the contrary, the model treats the teacher’s framing and repair work as part of the feedback event. Nor does it cover every use of AI in sport. Systems used solely for workload surveillance, talent identification, elite tactical analysis, diagnosis, or clinical rehabilitation raise related but distinct motivational and ethical questions.

### 2.2 Why PE is a distinctive feedback setting

Three characteristics of PE alter the meaning of feedback. First, the object of feedback is embodied. A learner does not encounter only a representation of performance; the learner has also felt the movement, observed its consequences, and may have a pre-reflective sense of timing, balance, effort, or discomfort. Felt experience can be mistaken, especially for novices, but it remains relevant evidence. AI feedback therefore enters a relation among quantified output, visual observation, task outcome, and bodily sensation.

Second, movement performance is often socially visible. PE places developing competence in front of peers more routinely than many academic subjects. Social comparison in PE can relate to engagement or disaffection depending on its referent, purpose, and relation to motivation (Barnes and Spray, 2013; Spray et al., 2023). In school PE, social physique anxiety is associated with motivational regulation and avoidance when learners feel their bodies are open to evaluation (Cox et al., 2011). AI does not create visibility, but persistent scores, replayable video, and rapid ranking can change its scale and consequences.

Third, AI feedback is embedded in pedagogical authority. An output may be introduced as one fallible source of evidence or as a machine-certified fact. Teacher language can explain why a cue matters and normalize error, or turn the output into pressure. Evidence from adolescent sport coaching associates the perceived style of corrective feedback with motivational and well-being responses (Mouratidis et al., 2010); this is neighboring evidence, not a university-PE study. School-PE research also connects perceived feedback, challenge, and threat with intrinsic motivation (Koka and Hein, 2003, 2006). These associations motivate attention to the instructional relationship without establishing the proposed causal sequence for AI feedback.

These features identify the focal setting rather than mechanisms unique to it. University PE combines assessed learning, repeated bodily performance, peer audiences, and institutional responsibility for student records. The same motivational processes may operate elsewhere, but different stakes, expertise, relationships, and safety constraints can change their strength. Claims of generality to coaching, rehabilitation, or other educational levels therefore require comparative tests.

### 2.3 Five outcome domains and their timescales

We distinguish five outcome domains throughout the model. System engagement concerns interaction with the technology, such as consultation or requests for feedback. Acquisition performance concerns execution during supported practice. Retention and transfer concern persistence after a delay and adaptation to a changed task or setting, respectively, with feedback conditions specified. Affect and well-being form a fourth domain: event-level anxiety or enjoyment should be distinguished from well-being assessed across a learning unit. Continued participation concerns voluntary physical activity beyond the instructional requirement and is the fifth, distal domain. Timescale is an attribute of measurement, not an additional outcome category. Need experiences and self-regulatory appropriation are proposed mechanisms and must not be relabeled as evidence of successful learning.

These outcomes can diverge. A learner may repeatedly consult a system yet practice poorly. A public rank may increase effort and immediate performance while reducing enjoyment. A correction may improve a trained movement but fail to transfer when the device is absent. Conversely, a learner may cease using the system because its monitoring function can be performed independently, not because the intervention failed. Work on digital behavior-change interventions similarly distinguishes engagement with an intervention from the engagement required for the target behavior and its outcomes (Yardley et al., 2016; Perski et al., 2017). SDT-informed analyses of behavior-change technologies warn that optimizing interaction with technology can displace the more important aim of internalizing the target behavior (Alberts et al., 2026; Gerstenberg et al., 2025). A motivational account of AI feedback must therefore explain not simply whether learners use the feedback, but what capability and reason for action remain when it is removed.

## 3 Theoretical resources and the unresolved link

### 3.1 Functional significance and psychological needs

SDT differentiates autonomous motivation—acting with volition or personal endorsement—from controlled motivation, in which behavior is organized by external demands or internal pressures (Ryan and Deci, 2020). It also proposes that the satisfaction of autonomy, competence, and relatedness supports high-quality motivation and well-being, whereas need frustration has distinct maladaptive correlates (Vansteenkiste et al., 2020). These distinctions are useful for AI feedback because a positive behavioral response does not reveal its motivational basis. Faster compliance with a correction can result from curiosity and growing competence, fear of public failure, desire to satisfy the teacher, or dependence on the next score.

Cognitive evaluation theory, a mini-theory within SDT, provides the immediate conceptual starting point. External events do not carry one invariant motivational effect. Their *functional significance* depends on whether they are interpreted primarily as informational—supporting a sense of effectiveness while preserving volition—or as controlling—pressuring the person toward a prescribed response. The same broad category of event can have different effects, which is why rewards and feedback should not be classified by form alone (Deci et al., 1999; Ryan and Deci, 2020). In sport, autonomy-supportive models similarly emphasize the informational delivery of competence feedback within a relationship that acknowledges the athlete’s perspective and minimizes control (Mageau and Vallerand, 2003).

Competence feedback is therefore not motivationally beneficial simply because it is accurate or positive. Competence must be experienced within a context that permits agency. A precise indication of failure without an attainable next step may make inadequacy clearer rather than support effectiveness. Likewise, nominal choice need not support autonomy if all options remain unintelligible or if questioning the output is prohibited. PE findings reinforce the relational character of these processes. Across a large meta-analytic evidence base, teacher support is associated with autonomy and competence, while teacher and peer support are similarly associated with relatedness, and autonomous motivation is associated with adaptive PE outcomes (Vasconcellos et al., 2020). Observational work further distinguishes need support and structure from control and chaos rather than treating instruction as a single good–bad continuum (Van Doren et al., 2025; Teraoka et al., 2024).

Organismic integration theory adds a temporal issue. Many movement-learning tasks are not intrinsically enjoyable at every moment. A student may initially practice because the course requires it. The important question is whether the rationale and regulation of practice become understood and personally endorsed. Internalization is not demonstrated by sustained clicks, repeated scores, or compliance during a graded unit. It is better indicated by increasing personal endorsement of the activity’s reasons and regulation, assessed separately from the capacity to perform or self-monitor without a device. This distinction has become prominent in analyses of behavior-change technologies, which contrast engagement-focused design with design that supports internalization and decreasing reliance on the technology (Alberts et al., 2026; Gerstenberg et al., 2025).

SDT already explains why feedback can have different functional significance. The present task is to specify how that established distinction can be investigated in AI-supported PE without defining meaning retrospectively from need satisfaction or performance. We therefore distinguish an appraisal of what the output communicates or demands from the subsequent experience of volition, effectiveness, or connection during action. This temporal distinction is a proposed analytical decomposition of the feedback event, not a claim that SDT overlooks interpretation.

### 3.2 Feedback uptake and self-regulation

Feedback theory locates the learner more actively in the process. Butler and Winne (1995) describe self-regulated learning as a recursive sequence in which learners monitor performance, compare it with goals, generate internal feedback, and adapt strategies. External feedback becomes useful when it enters and changes that internal process. It is not effective merely because it is present.

Feedback literacy offers a complementary account of uptake. Learners need to appreciate feedback, make judgments about quality and work, manage affective responses, and take action (Carless and Boud, 2018). Recent scholarship on AI-assisted feedback similarly treats perceptions, emotions, actions, and literacies as connected layers of the feedback process (Yang et al., 2026). Systematic evidence suggests that learners recognize benefits in AI-generated feedback but vary in their preferences, trust, and judgments of AI and human sources (Öztürk and Çebi, 2026). A meta-analysis of trust in educational AI further identifies technological performance as a strong antecedent while showing that user experience, individual differences, feedback level, and task type also matter (Cui et al., 2025). Accuracy matters, but it is interpreted within a broader relation.

Motor learning adds two cautions. First, the feedback that optimizes performance during practice does not necessarily optimize retention. Second, learner control is not a sufficient mechanism. Some research reports motivational and feedback-processing differences under self-controlled feedback (Grand et al., 2015), while a meta-analysis reports performance and learning benefits (Jimenez-Diaz et al., 2021). Bias-corrective work nevertheless suggests that benefits have been exaggerated (McKay et al., 2025). The relevant capacity may be not choice over when to receive feedback, but judgment about what evidence is needed, what it means, and how to act on it. The OPTIMAL theory of motor learning places autonomy support and enhanced expectancies within a wider account of attention and goal-action coupling (Wulf and Lewthwaite, 2016), but strong tests still need to separate immediate expectancy effects from durable self-regulatory change.

Augmented-feedback research provides a more specific basis for separating supported performance from learning. The guidance hypothesis proposes that frequent external feedback can guide practice while limiting the development of independent error detection (Salmoni et al., 1984). Experiments on reduced relative frequency and fading show why performance during practice cannot stand in for retention (Winstein and Schmidt, 1990; Wulf and Schmidt, 1989). Bandwidth feedback supplies augmented information when error exceeds a criterion, which changes both the schedule and the information carried by its absence (Sherwood, 1988). These findings do not imply that less feedback is always better. Reviews emphasize interactions with task complexity, learner expertise, and feedback content (Sigrist et al., 2013). For the present model, they establish a rival and complementary explanation: differences after AI withdrawal may arise from feedback scheduling or information processing rather than motivational meaning. Tests of the motivational pathway must therefore match or explicitly manipulate frequency, fading, and error-contingent thresholds instead of leaving them confounded with framing or contestability.

We use self-regulatory appropriation to describe a proposed process in which monitoring and adjustment initially organized by external feedback become demonstrable without the same support. It is not a general trait or a synonym for good learning. The relevant transition includes generating an evaluative criterion, checking a discrepancy, selecting or revising a cue, and seeking additional evidence when appropriate. Feedback literacy concerns capacities for engaging with feedback; self-assessment and metacognitive monitoring are component operations; SDT internalization concerns the endorsement of regulation. Appropriation would add value only by identifying the within-person transition from externally cued to independently initiated use of those operations. Its indicators must be process measures collected before the retention or transfer outcome, rather than successful transfer itself. If established self-regulation and feedback-literacy measures explain that transition equally well, the new label should be merged with them rather than retained as an additional construct.

### 3.3 Embodied evidence and calibrated trust

AI feedback in PE is unusual because the learner can compare data with an ongoing bodily experience. Conceptual critiques of fitness tracking argue that prolonged quantification may objectify the body and displace attention from felt sensations that are important to adaptive physical activity (Toner, 2018). Such arguments should not be converted into evidence that bodily feeling is always accurate or that quantification is inherently alienating. Novices can misperceive their movements, and external visual or kinematic information can reveal errors that feeling alone does not detect. Digital video, slow motion, and posture analysis can support self-assessment and motor learning, especially when criteria and guidance are clear (Müller and Wagner, 2026).

Calibrated trust means that judgments of an output’s dependability and decisions to rely on it track independently established task validity and uncertainty. It is not high average trust. A learner who accepts both sound and erroneous cues has high reliance but poor discrimination; a learner who rejects both has low reliance and similarly poor discrimination. A test should score each output against an independent criterion, obtain a probability judgment of its correctness, and record acceptance, checking, deferral, or rejection. Probability calibration can be evaluated by comparing estimated and observed correctness, while conditional reliance distinguishes acceptance of valid from invalid advice. The two should be reported separately because a learner may distrust a cue yet follow it under grading pressure. Cases with uncertain criterion validity should allow deferral rather than treating either trust or rejection as correct by definition.

This comparison is especially important when an AI output has high apparent authority but low explainability. A generic human cue may be recognized as one person’s judgment; a numerical output can look definitive even when its training data, thresholds, or confidence are invisible. Reviews of AI in PE identify accuracy, fairness, privacy, and changing teacher–student relations as unresolved concerns (Wang and Wang, 2024), while AI-feedback research notes limited algorithmic transparency (Ba et al., 2025). Trust should therefore be treated as a calibrated, revisable judgment rather than an outcome to maximize.

### 3.4 The unresolved link

SDT supplies functional significance and need-based motivation; feedback literacy and self-regulated learning supply active judgment and uptake; motor-learning research distinguishes supported acquisition from durable learning. The present model brings these resources to a specific conjunction: algorithmic output is interpreted through a teacher, a peer audience, and bodily or task evidence within assessed instruction. These mechanisms are not exclusive to university PE. Coaching and rehabilitation may contain the same elements. University PE is the focal configuration because developing bodily competence is often visible to peers, participation and assessment are institutionally organized, and learners differ widely in skill and prior activity. Whether this configuration changes the strength of the proposed relations requires comparative evidence.

The model’s additional explanatory commitment is a set of separable event-level relations. It predicts that a right to challenge an output can matter when information access and teacher contact are unchanged, that named disclosure can alter controlling meaning when the learner receives the same reference information, and that unresolved evidence conflict can interrupt action even without a global rejection of the system. These are specified applications that make the integrated account more testable; they are not effects that SDT or self-regulated learning could never accommodate. The integration is useful only if these relations explain more than technical quality, general teacher support, and established learner capacities alone.

SDT-informed behavior-change technology accounts distinguish engagement with a technology from internalization of the target behavior (Alberts et al., 2026; Gerstenberg et al., 2025). We retain that distinction while concentrating on the shorter feedback episode and the transition to independent regulation of a movement task. This focus yields a restricted contribution: a causal hypothesis about the organization and interpretation of AI feedback, with explicit measurement and comparison requirements. It does not establish priority over all existing applications or replace the broader theories from which its predictions are developed.

## 4 A motivational-meaning model of AI feedback

### 4.1 AI-specific properties

Technical accuracy concerns agreement with an independently justified criterion for the intended task, person, and movement context. Validity also requires that the estimated feature matters for the intended instructional judgment. A precise joint-angle estimate is not automatically a valid assessment of skill. Perceived accuracy is the learner’s belief about correctness and must be measured separately. A fair comparison should document the model version, task, camera arrangement, output latency, cue wording, score, confidence display, and feedback frequency. ‘Technically equivalent’ means that these properties are matched or experimentally controlled for the comparison at issue; it does not mean that an identical score is equally valid for different bodies, assistive equipment, or movement adaptations. Criterion uncertainty and subgroup performance must therefore be reported rather than hidden inside a single accuracy value.

Five properties make AI a theoretically revealing case. First, automated output can carry *apparent epistemic authority*. Second, the basis of that authority may remain *opaque*. Third, movement can be represented with unusual *granularity and persistence*. Fourth, feedback can be *rapidly personalized and repeated*. Fifth, it can be *circulated and compared* at low cost. A teacher can use these properties to make progress visible and action intelligible. The same properties can create continuous surveillance, amplify a deficit, or make a questionable inference difficult to contest.

### 4.2 Three conditions of situated interpretation

*Pedagogical framing* concerns how the output’s purpose, reference standard, rationale, action possibilities, and authority are organized. Mastery framing treats the output as provisional evidence for an attainable next step and communicates uncertainty where appropriate. Controlling framing treats it as a demand, a basis for conditional regard, or a final verdict. Framing includes what the teacher says, but it is not reducible to interpersonal warmth. A friendly teacher can still present an algorithmic score as non-negotiable, and a demanding lesson can preserve agency through clear rationale and meaningful challenge.

Social visibility concerns who can see the output, how it is compared, and what follows from that comparison. Private, self-referenced review differs from named leaderboards, public replay, or norm-referenced grading. Visibility is not uniformly harmful. Voluntary sharing in a cooperative climate can support relatedness and observational learning. The central distinction is between visibility that serves collective inquiry and evaluative exposure that makes developing competence consequentially comparable. Evidence on social comparison and ability exposure in PE supports attending to the referent, motive, belonging, and anticipated judgment rather than classifying all peer observation in one way (Barnes and Spray, 2013; Spray et al., 2023; Wang, 2026).

Embodied verification concerns access to opportunities for comparing an AI output with felt movement, task effects, replay, and knowledgeable interpretation. It is a procedure for coordinating fallible evidence, not a claim that sensation outranks measurement. We distinguish access to verification resources from their effective use, which depends partly on skill and feedback or AI literacy. Contestability is a related but separable institutional permission: the learner can question an output, request a review, and decline or revise a recommendation on stated grounds without disproportionate penalty. Replay or an explanation may make a cue understandable without giving the learner that permission. This separation permits tests of information access, human support, and the right to challenge as different components.

The three conditions are selected because they answer different questions about the same event: what is this output for, who can judge me through it, and what evidence can I use to assess it? They are a parsimonious organization of the focal mechanism, not an exhaustive or uniquely necessary taxonomy. Explainability, confidence information, and technical accuracy are system inputs; AI and feedback literacy are learner resources; perceived accuracy is an event appraisal; and general teacher climate is a broader contextual influence. Each can affect one or more focal conditions, and each remains a potential rival predictor. The model should be expanded or simplified if another organization explains the evidence better.

The inputs operate at different levels. System characteristics determine what can be estimated, explained, stored, or replayed. Teacher enactment determines how a particular output is introduced and discussed. Assessment arrangements determine whether following the output affects a grade or other consequence. Institutional data practices determine who can access the record, how long it persists, whether it can be corrected, and what consent covers. For example, a display seen only by the learner during class may still be experienced as evaluative if it will later enter a permanent assessment record. General teacher climate can influence all three event conditions, but cannot substitute for observing their enactment. Figure 1 makes these contextual inputs explicit without adding a separate psychological mediator for each administrative feature.

**Figure 1:**
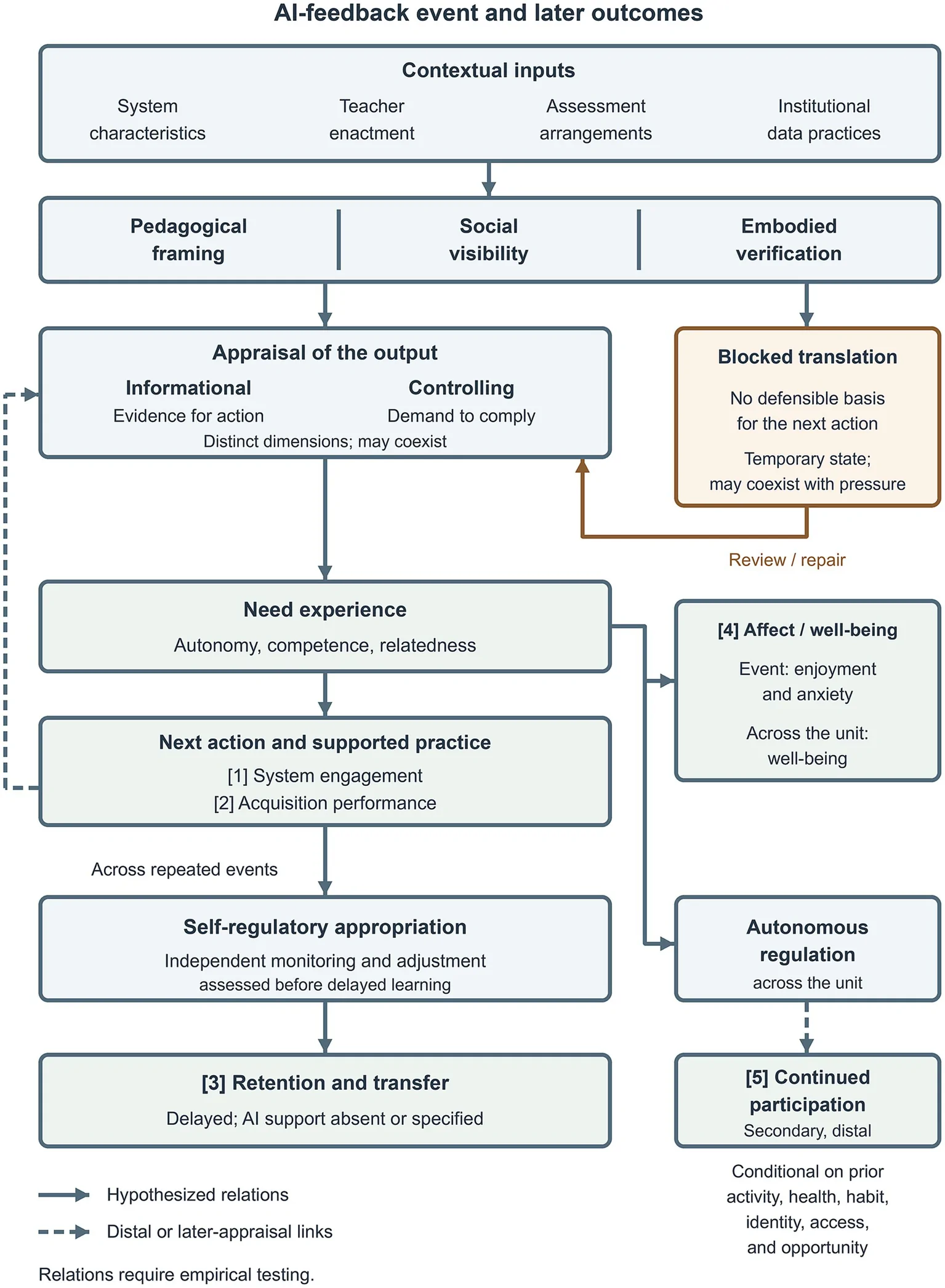
Hypothesized sequence of an AI-feedback event and its extension across time. System characteristics, teacher enactment, assessment arrangements, and institutional data practices organize pedagogical framing, social visibility, and embodied verification. Informational and controlling appraisals are distinct from subsequent need experiences and may coexist. Blocked translation is shown separately as a temporary impediment to selecting an evidential basis for action, with a review route back to appraisal; it is not a third motivational meaning. Repeated events may contribute to self-regulatory appropriation, measured before delayed learning outcomes. The five outcome domains are system engagement, acquisition performance,retention and transfer, affect and well-being, and continued participation. Solid arrows indicate hypothesized within-event or repeated-event relations, not established causal effects. Dashed arrows mark the secondary distal participation pathway and influences from earlier performance on later interpretation. Prior activity, health, habit, identity, access, and opportunity constrain continued participation. The diagram is schematic; direct, reciprocal, and alternative paths require empirical comparison.

### 4.3 Motivational meaning and a blocked translation state

Informational meaning is an appraisal that an output offers evidence about a task discrepancy and a possible basis for choosing an adjustment. It is measured by what the learner understands the message to say and permit, not by reporting ‘I feel competent’ or ‘I feel autonomous.’ An item might ask whether the message was presented as evidence to examine before choosing a next step. Such items are provisional and require validation. Negative corrective feedback can be informational. Associational evidence from adolescent sport coaching is consistent with that possibility, but does not validate the present event-level decomposition in university PE (Mouratidis et al., 2010).

Controlling meaning is an appraisal that compliance with the output is expected to avoid an evaluative consequence, sanction, or loss of standing. A candidate indicator asks whether following the recommendation was understood as a requirement for avoiding a penalty, rather than whether the learner felt anxious or incompetent afterward. Informational and controlling appraisals can coexist: a learner may recognize useful evidence and also perceive pressure. They should initially be measured as distinguishable dimensions, not forced into mutually exclusive learner categories. Subsequent need satisfaction or frustration concerns the experienced quality of action and relationships, which can be influenced by prior skill and context as well as by the appraisal.

Blocked translation denotes a temporary inability to establish a defensible evidential basis for the next action after encountering conflicting information. A discrepancy between AI output and other evidence is a proposed antecedent, not by itself proof of a blockage. Candidate indicators include inability to identify which evidence should guide the next attempt, unresolved oscillation among incompatible cues, and delayed strategy selection despite understanding the task. These should be observed before coding later compliance, rejection, or performance. Blockage is not a third motivational meaning and does not require the absence of controlling pressure: a learner can be unsure what is warranted while feeling compelled to comply. Confusion, epistemic uncertainty, distrust, and need frustration are plausible simpler explanations. The state should be retained only if it can be distinguished from them and predicts a specified next-step process beyond them.

The blocked state can resolve when relevant evidence is interpreted, an error is corrected, or a defensible decision to defer is made. The ensuing output may be appraised as informational, controlling, or both. Repeated public exposure and prior success can also change later appraisals. Figure 1 therefore separates a provisional temporal sequence within an event from feedback across events. It does not assert that interpretation, need experience, and action occur in perfectly isolated psychological stages.

A discriminating measurement sequence begins with the output and observed context, followed by an immediate appraisal of its evidential content and demands, then a separate assessment of need experience, and finally observation of the next action, affect, and performance. A brief pre-action need assessment can distinguish appraisal from the effect of the next performance; a later measure can capture change during action. Informational items must not ask whether the learner feels effective, controlling items must not be anxiety items, and blockage items must not be defined by later poor performance. Baseline need states and prior performance remain possible causes of appraisal, while later performance can update the next event. The proposed ordering is thus a testable sequence with reciprocal alternatives, not a definition that guarantees mediation.

Table 1 defines the central constructs without substituting downstream outcomes for their indicators. Supplementary Table S1 compares blocked translation and appropriation with their nearest alternatives, including antecedents, candidate indicators, and criteria for incremental value. None of the proposed items constitutes a validated scale.

**Table 1:** Central constructs and independent indicators.

Construct	Definition	Independent indicator or distinction
AI-feedback event	An episode in which AI-derived movement information is encountered within instruction.	Record the output, task, timing, teacher involvement, audience, and assessment use.
Pedagogical framing	How purpose, reference, rationale, and authority are communicated.	Code the instructional message; separately check the learner’s perception of its purpose.
Social visibility	Who can access identifiable feedback and what comparison or consequence is attached.	Observe actual disclosure, access, and persistence; hold the learner’s own reference information constant in visibility tests.
Embodied verification	Access to opportunities to compare output with bodily, task, visual, and human evidence.	Document accessible evidence and opportunities for reattempt or review; distinguish access from the skill to use it.
Informational appraisal	Interpreting output as task evidence and a possible basis for selecting an adjustment.	Ask what evidence and action options the message communicates; do not measure this with competence or autonomy satisfaction items.
Controlling appraisal	Interpreting compliance as required to avoid an evaluative consequence.	Ask what demand and consequence the message communicates; keep anxiety and need frustration as separate measures.
Blocked translation	A temporary inability to establish a defensible evidential basis for the next action.	Assess evidence-selection uncertainty and unresolved strategy selection before the next attempt; it is not a third meaning.
Self-regulatory appropriation	A transition from externally prompted to independently initiated monitoring and adjustment.	Code criterion generation, evidence selection, and strategy revision during prompt reduction, before separate retention and transfer tests.
Calibrated trust and reliance	Trust judgments and reliance that track independently established validity and uncertainty.	Compare judged with observed correctness; separately estimate reliance on valid versus invalid cues and justified deferral.

All indicators are proposals for validation; Supplementary Table S1 compares the two provisional process constructs with their closest alternatives.

*Proposition 1 (separable contextual effects). P1a: With output content, reference information, and audience held constant, mastery-oriented rather than pressuring framing will increase informational appraisal and reduce controlling appraisal. P1b: With the same individual output, norm reference, and grading consequence, named disclosure to peers rather than private display will increase controlling appraisal. P1c: When independently established output–criterion disagreement is presented, withholding an otherwise available verification opportunity will increase next-step evidence-selection uncertainty relative to providing that opportunity; this difference is predicted to be smaller for criterion-concordant output. These are separate primary contrasts, not a prediction that every combination improves every outcome.*

Figure 1 distinguishes contextual inputs, interpretation, need experience, and subsequent action. System characteristics, teacher enactment, assessment arrangements, and institutional data practices organize the three interpretive conditions. Event appraisals are measured before the next attempt; need experience is assessed separately; action, affect, and acquisition are then observed. Across repeated events, appropriation is a candidate mechanism linking interpretation with retention and transfer. The same five outcome domains introduced in Section 2.3 are retained. The continued-participation pathway is dashed because it is distal, secondary, and contingent on influences beyond classroom feedback.

## 5 Mechanism pathways and propositions

### 5.1 Competence: from an objective-looking score to actionable progress

AI feedback appears especially well suited to competence support. It can identify a discrepancy that a learner or teacher misses, display gradual improvement, and tailor a cue to the current attempt. The positive interpretation of these functions is visible in reviews of digital formative assessment and AI-supported personalized feedback (Ba et al., 2025; Müller and Wagner, 2026; Wang W. et al., 2026). Yet the inference from more information to greater competence is incomplete.

Competence concerns experienced effectiveness in interaction with an environment, not the quantity or precision of information received. Three conditions intervene. First, the criterion must be valid for the task and learner. An accurate estimate of an irrelevant feature does not support useful competence. Second, the discrepancy must be connected to an attainable challenge. Repeatedly documenting a gap that the learner cannot yet close can make failure more legible without making improvement more possible. Third, feedback must provide an intelligible action path. “Your trunk angle is 12 degrees outside the reference range” may be specific but not actionable; “on the next attempt, keep the chest facing the landing point and compare the resulting balance” links evidence to a controllable adjustment.

This distinction also clarifies why positive feedback is not always informational and negative feedback is not always controlling. A high score may shift attention toward preserving status or beating peers. A corrective message can support competence if it identifies meaningful progress and a next action while treating error as revisable. PE studies relating feedback, challenge, and threat to intrinsic motivation are consistent with this functional distinction (Koka and Hein, 2003, 2006). Likewise, a systematic review of digital formative assessment found that students’ self-assessment benefited from clear criteria and structured teacher guidance, while autonomy alone was not uniformly decisive (Müller and Wagner, 2026). The relevant pedagogical achievement is not maximal freedom from guidance but structure that increases the learner’s capacity to judge and act.

The pathway is expected to be dynamic. Early in learning, a learner may need a narrow external cue and model. With practice, competence support should become visible in increasingly accurate self-assessment, selection of relevant information, and adjustment without a prompt. If the system continues to prescribe each response after the learner can regulate it, information can become redundant or controlling. If output frequency is reduced before the learner has a usable internal reference, the result may be chaos rather than autonomy.

*Proposition 2 (intelligible action). For a valid cue describing a task discrepancy that is attainable within the learner's current capability, adding an intelligible next-action explanation will increase subsequent competence satisfaction relative to a matched description of the discrepancy alone. The primary contrast holds score, valence, specificity, practice dose, and feedback frequency constant. Attainability is assessed independently before manipulation rather than inferred from a favorable response.*

This proposition separates knowing the size of an error from understanding an available adjustment. A manipulation check should test comprehension of the proposed action, while the need measure concerns subsequent experienced effectiveness. Failure to distinguish these measures would make the test circular. The account would be weakened by a precisely estimated absence of the predicted competence difference despite successful manipulation and adequate measurement. Any effect on retention is a separate downstream question, not part of the definition of actionable feedback.

### 5.2 Autonomy: from nominal choice to contestability

AI-supported learning often operationalizes autonomy as choice: whether to request feedback, which display to view, or when to repeat a task. Choice can be useful, but it is neither necessary nor sufficient for autonomy. A learner may choose among options that are poorly understood, select feedback to manage impressions, or feel responsible for complying with an output that cannot be questioned. Evidence on self-controlled feedback in motor learning is mixed once reporting bias and statistical power are considered (McKay et al., 2025), suggesting that the motivational and learning consequences of “control” cannot be inferred from a choice button alone.

Contestability is the effective right to challenge and obtain reconsideration of an output without a disproportionate social or grading penalty. It may require institutional arrangements, but it is not defined as the entire package of replay, explanation, confidence information, teacher attention, and alternative evidence. Those resources can be present in both contestable and non-contestable conditions. The theoretical claim is that understanding a recommendation and having a legitimate say in its use are different properties of instruction.

An isolating test would provide both groups with the same replay, uncertainty information, alternative evidence, teacher-contact time, and choice over display timing. The groups would differ only in whether a documented challenge can lead to a reviewed correction or an acceptable decision to defer. A separate factor could vary explanation or verification resources. This design distinguishes a right to challenge from receiving better information or more personal support. The comparison should be conducted in low-stakes practice or a simulation, without creating an unfair assessment procedure.

The challenge route must be credible rather than a cosmetic permission. Where exercising it produces additional information or corrective teacher feedback, the estimated effect belongs to that specified review procedure and should not be attributed to permission alone. A matched review in the comparison condition or a component design can help distinguish these possibilities. Both designs should record the information actually received and the responses to a challenge.

Contestability should not be confused with unrestricted learner preference. Safety constraints, assessment standards, and limited class time can justify structure. Autonomy support concerns how such constraints are communicated and whether learners can understand their relevance, express a perspective, and exercise judgment within them (Ryan and Deci, 2020). A system may also correctly override a learner’s initial belief. What matters is whether the correction can be incorporated through reasons and evidence rather than accepted as an opaque command.

*Proposition 3 (contestability). When technical information, verification resources, teacher contact, and nominal feedback choice are held constant, an effective right to challenge and request revision of an output will increase subsequent autonomy satisfaction. Across independently valid and invalid outputs, it will also improve discrimination in reliance, expressed as greater appropriate reliance on valid than invalid advice. The autonomy contrast and the quality-by-contestability interaction are separate preregistered tests.*

The manipulation check concerns whether a challenge is understood as permissible and consequential. It must be separate from the need measure and from actual use of the challenge route. A rise in undifferentiated acceptance would not support the calibration prediction. A contestability effect that disappears when information and teacher support are matched would instead favor those components as explanations. Statistical models should distinguish reliance from trust judgments and allow justified deferral when criterion evidence is inconclusive.

### 5.3 Relatedness and evaluative exposure

AI feedback may appear impersonal, but its meaning is profoundly social. The output is introduced by someone, seen by an audience or kept private, and positioned within a relation of assistance, assessment, or comparison. Relatedness concerns feeling cared for and significant to others; need frustration includes rejection and exclusion (Vansteenkiste et al., 2020). In PE, where bodies and skills are visible, a feedback display can communicate whether difficulty is treated as a normal part of learning or as a public marker of deficient ability.

Social visibility is therefore not a simple exposure count. Its motivational function depends on audience, reference, voluntariness, and consequence. A private, self-referenced trajectory can help a student see progress. A voluntarily shared replay can support peer dialogue. An anonymous class pattern can normalize common errors. In contrast, a named leaderboard, public error flag, or repeated replay of a poor attempt can make feedback norm-referenced and identity-relevant. Social-comparison research in PE indicates that consequences vary with comparison targets, motives, and self-determined motivation (Barnes and Spray, 2013; Spray et al., 2023). Research on social physique anxiety further shows why bodily display can be related to avoidance and effort (Cox et al., 2011).

AI can intensify evaluative exposure in three ways. It can give comparison an appearance of neutrality, preserve outputs that would otherwise be transient, and make rankings rapidly scalable. Learners who might dismiss a peer comment can experience a system score as official. Repeated, fine-grained records can also turn ordinary variability into a stable profile of ability. Recent correlational evidence in university PE links ability exposure to evaluation anxiety and lower psychological well-being (Wang, 2026). Yet visibility is not uniformly harmful. In a cross-sectional study of university students who used physical-activity app leaderboards, leaderboard use was associated with more social comparison and activity, had no direct association with stress, and was indirectly associated with lower stress through activity-related paths (Wang S. et al., 2026). The authors also emphasized heterogeneous effects and the possible burden of persistent unfavorable comparison. Taken together, these studies make visibility a plausible moderator rather than a cosmetic design choice, while cautioning against a simple public-equals-harmful rule.

For the primary prediction, evaluative visibility means named disclosure of the same individual performance information to peers. The comparison keeps the learner’s own norm-referenced information and grading consequences unchanged. Baseline perceived competence is the primary proposed moderator, measured before feedback: the predicted pressure effect is larger at lower perceived competence. Social physique anxiety is an additional prespecified moderator only where the design can estimate that interaction with adequate precision. Voluntary cooperative sharing is a different instructional arrangement and should be examined separately, not invoked afterward to reverse the primary prediction.

*Proposition 4 (evaluative visibility). In assessed university PE, named peer disclosure of an individual's AI feedback will increase controlling appraisal and subsequent evaluation anxiety relative to private display of the same feedback, with the learner's norm reference and grading consequences held constant. The controlling-appraisal effect is predicted to be larger at lower baseline perceived competence. A null or reversed contrast under these specified conditions would weaken the proposition; benefits of voluntary cooperative sharing would address a separate comparison.*

A test should verify who actually saw the output, whether the learner anticipated peer judgment, and whether reference information and assessment consequences were matched. Anxiety is measured after the appraisal rather than embedded in its definition. The main disclosure contrast and competence interaction should be specified before data collection. Public ranking with altered stakes, cooperative replay, and private self-reference should not be combined into a single manipulation and then interpreted as a pure visibility effect.

### 5.4 Body–data discrepancy and blocked translation

Every feedback system can be wrong, and every learner can misperceive a movement. AI makes the relation theoretically important because the output may combine high apparent precision with low interpretability. A learner can see “87% incorrect” without knowing the confidence interval, normative sample, camera limitation, or criterion weighting. When the score conflicts with bodily sensation or task success, a feedback event becomes an epistemic problem: what should guide the next attempt?

One possibility is productive recalibration. The learner replays the movement, attends to a previously unnoticed feature, tests a cue, and finds that the revised movement improves both the task outcome and felt control. Here, discrepancy becomes informational through verification. A second possibility is justified correction of the system: camera occlusion, atypical body proportions, assistive equipment, or a task variation produces a misleading classification. If the learner can demonstrate the mismatch and the teacher treats the output as fallible, contesting the system can support competence and autonomy.

A blockage is possible when conflicting evidence leaves no defensible basis for choosing the next adjustment. The immediate prediction concerns unresolved evidence selection, not a catch-all combination of dependence and rejection. Subsequent behavior depends on conditions that must be specified separately: high anticipated penalties may encourage compliance despite unresolved doubt, whereas a credible alternative and low exit cost may permit rejection of the disputed cue. These branches are secondary hypotheses. They do not define the state and cannot both be counted as confirming the same undirected prediction.

The proposal connects concerns about accuracy and trust with the embodied character of PE. Research on tracker use, educational AI trust, and the phenomenology of quantification offers adjacent evidence (Nuss and Li, 2021; Cui et al., 2025; Toner, 2018). None establishes blocked translation in AI-supported university PE. The term is a provisional description whose value depends on discriminant measurement and a specified prediction about evidence selection, beyond general confusion, perceived inaccuracy, epistemic uncertainty, and trust.

Teachers may help resolve a discrepancy by distinguishing model error from movement error and directing attention to independently relevant evidence. To test repair rather than general interpersonal support, conditions should match teacher-contact time, respectful language, and available display information. The active component is a structured examination of the conflicting evidence and an opportunity to reconsider the disputed interpretation; whether this resolves the mismatch is assessed as an outcome. Correction of a system error and correction of a learner’s misperception should both be possible outcomes; repair is not defined as greater agreement with the AI.

*Proposition 5 (discrepancy and repair). Independently verified disagreement between an AI output and relevant task evidence will increase evidence-selection uncertainty relative to concordant output when the discrepancy cannot be reviewed. Providing a discrepancy-specific review will reduce the duration or repeated occurrence of that uncertainty more strongly for discordant than concordant output. This quality-by-review interaction, rather than either dependence or rejection, is the primary prediction.*

The first test should use naturally occurring documented errors or recorded, non-graded scenarios in which no participant acts on unsafe advice. Any study that deliberately presents misleading feedback to participants performing a task would require prior ethics approval, a justification that a non-deceptive alternative cannot answer the question, the minimum necessary deception, and a low-risk task. Consent arrangements must be approved in advance; grades and access to instruction must be unaffected; withdrawal must carry no penalty. A qualified instructor should monitor distress and safety, stop the procedure when needed, correct false competence or performance impressions, and conduct prompt, careful debriefing with an opportunity to ask questions and, where approved, withdraw affected data. Manipulation checks and a post-debrief welfare check must not expose participants to further misleading practice. These are requirements for possible future studies, not procedures performed for this theoretical article.

### 5.5 Appropriation separates performance from transfer

The model’s most important temporal distinction concerns what remains after feedback is removed. Informational meaning is expected to support need satisfaction, but favorable feeling alone does not produce learning. In the proposed regulatory pathway, learners convert external output into an internal reference and a repertoire of monitoring and adjustment. A student who can state “I will attend to the pressure shift before landing and use balance on the next attempt as evidence” has acquired more than a score. The learner has a cue, a comparison process, and a decision rule that can travel to a later task.

Controlling feedback can produce a different trajectory. A mandatory target may focus effort and improve acquisition while leaving action dependent on an external instruction. This possibility is consistent with consumer research in which measurement increased activity quantity while reducing enjoyment and later engagement (Etkin, 2016). That evidence does not establish a PE mechanism. The present prediction is that the quality of regulation should help explain what persists after support is withdrawn, beyond the immediate amount of practice. Acquisition and independent regulation must therefore be measured separately.

Blocked translation may impede the development of an independent reference when learners cannot determine which evidence warrants an adjustment. This is a hypothesis about a process, not evidence that every episode causes dependence or withdrawal. A successful review could instead improve later evidence selection. Studies should record whether learners subsequently generate a criterion, compare evidence, and initiate an adjustment without a prompt before evaluating whether those processes explain later learning.

Retention and transfer are therefore more diagnostic than immediate performance. Retention asks whether a movement change persists after time and feedback removal. Transfer asks whether the learner can adapt the skill or regulatory process in a changed setting. The recent review of digital formative assessment in PE is encouraging about performance and self-assessment but explicitly limited by the small number of retention tests and the controlled nature of many studies (Müller and Wagner, 2026). A motivational-meaning model predicts that acquisition effects can converge while delayed outcomes diverge.

Continued participation is a secondary pathway that requires a different design. Associations between autonomous motivation and physical activity have been reported across school and developmental contexts (Hagger et al., 2003; Hutmacher et al., 2020; Courtney et al., 2021; Owen et al., 2014). They do not establish that university-PE AI feedback causes later activity. Reviews of motivation interventions and maintenance theories further caution that behavior depends on processes and circumstances beyond initial motivation (Ntoumanis et al., 2021; Kwasnicka et al., 2016; Rhodes and Sui, 2021). Prior activity, health, habit, identity, access, time, and available opportunities may dominate any classroom contribution. A longitudinal study must measure these factors and acknowledge remaining confounding; intentions and platform use cannot substitute for observed voluntary participation.

*Proposition 6 (appropriation and transfer). With task, practice dose, feedback schedule, and technical quality held constant, repeated informational rather than pressuring feedback arrangements will improve independently initiated monitoring and adjustment before support withdrawal. These process indicators will prospectively predict delayed retention and transfer assessed without the original AI support, beyond baseline skill and pre-existing self-regulation. Mediation through appropriation is a hypothesis requiring additional identification assumptions, not a consequence of observing an association.*

The strongest design randomizes the instructional arrangement, fixes practice opportunities, measures appropriation during a planned reduction of prompts, and subsequently tests retention and transfer using distinct tasks. Acquisition is measured as an outcome. Because it can be affected by the intervention, mechanically equating groups on post-intervention acquisition or adjusting for it in the primary causal analysis can distort the comparison. Acquisition-adjusted analyses may be reported as clearly qualified sensitivity analyses. The additional appropriation construct is weakened if it is indistinguishable from established self-regulation measures or provides no prospective information beyond them. Reverse paths from performance and need experience to later appraisal should also be examined.

### 5.6 Continued participation as a secondary hypothesis

*Proposition 7 (distal continued participation). Across a PE unit, repeated informational appraisals are hypothesized to predict voluntary post-course activity through more autonomous regulation, over and above baseline activity and measured opportunity. This is a secondary prediction to be tested at repeated follow-ups with health, habit, identity, access, and changing opportunities considered. If its prospective contribution is negligible once these factors are modeled, the participation pathway should be removed without treating the event-level or transfer account as thereby disproved.*

This hypothesis cannot be evaluated by enjoyment during a lesson, an intention to exercise, or continued use of an AI application. At minimum it requires temporally separated measures of the proposed classroom exposure, autonomous regulation, and subsequent voluntary activity. Observational adjustment reduces some alternative explanations but cannot establish that the classroom pathway caused later participation. A randomized intervention with adequate follow-up would provide stronger evidence, while still requiring attention to attrition, changing opportunity, and interference between students.

## 6 Rival explanations and boundary conditions

The model is not intended to absorb every determinant of AI-supported learning. Its value depends on outperforming simpler explanations under appropriate tests.

An *accuracy and personalization account* predicts that valid, specific, and individualized feedback improves outcomes because it reduces informational error. The motivational-meaning model accepts these properties as important inputs but predicts different responses to identical output under different framing, visibility, and verification conditions. A decisive test therefore holds technical quality constant. If technical properties explain outcomes fully, situated translation is unnecessary.

A *novelty or expectancy account* predicts short-term effort because AI is interesting or believed to be effective. Novelty may explain initial engagement and favorable self-reports, while teacher or researcher expectations may amplify them. Longitudinal designs should assess prior AI experience, credibility expectations, and change as novelty recedes. The focal model predicts that interpretive conditions continue to matter after these effects diminish.

A *performance-first account* reverses the proposed direction: accurate feedback improves performance, which then increases competence and motivation. This reciprocal path is plausible and is included in Figure 1. The focal claim is not that motivation always precedes performance, but that framing, visibility, and verification should influence meaning and regulation even when immediate performance is comparable. Trial-level temporal data and experimentally induced conditions are needed to separate these paths.

A *generic teaching-quality account* locates the effect in an autonomy-supportive, structured, and caring teacher rather than in AI feedback. General instructional quality is an important alternative. Studies should measure it, compare events within the same teacher, and manipulate AI-specific properties such as confidence disclosure and error contestability. The present model earns explanatory value only if apparent machine authority, opacity, and body–data discrepancy produce patterns beyond general teacher support.

Individual differences offer further alternatives and moderators. Baseline skill and self-efficacy can change both performance and interpretation. Feedback literacy and AI literacy can affect judgment and action. Social physique anxiety can intensify evaluative exposure, while prior exclusion or disability can make a supposedly neutral reference model identity-relevant. Cultural norms may change the meaning of teacher authority, public comparison, and disagreement. These factors should be measured and, where theoretically justified, modeled as moderators. The situated claim is compatible with person differences: it predicts that context systematically changes response distributions rather than making everyone respond alike.

The model has substantive boundaries. It addresses AI-augmented feedback for skill learning in university PE, not autonomous replacement of teachers, high-performance sport optimization, clinical rehabilitation, or generalized fitness surveillance. It is most applicable when movement can be attempted repeatedly and evidence can be compared. In high-risk skills, contestability cannot mean unrestricted experimentation; safety may require tighter structure and expert override. In less observable skills or activities in which aesthetic, tactical, or interpersonal judgment is central, the balance among quantitative, qualitative, and embodied evidence may differ. Access to reliable hardware, inclusive reference data, privacy protection, and accommodation for diverse bodies are preconditions for a fair test of the motivational model rather than secondary design details.

## 7 A research agenda for strong tests

### 7.1 Validate the meanings without defining them by outcomes

Construct development should begin with interviews and stimulated recall anchored to concrete, ethically obtained feedback events, followed by content review, cognitive testing, and separate exploratory and confirmatory samples. Informational appraisal concerns evidence and action options; controlling appraisal concerns an expected requirement and its consequences. Need satisfaction and frustration concern subsequent experience of volition, effectiveness, and connection. Candidate items must not repeat the same wording across these levels. Blocked translation is assessed separately as an episode of unresolved evidence selection. Supplementary Tables S1 and S2 specify candidate indicators and comparisons; they are a research agenda, not a newly validated instrument.

Measurement studies should compare a two-dimension appraisal model with alternatives allowing overlap, a single evaluative factor, or direct relations from established constructs to outcomes. A separate state measure for blocked translation should be tested against confusion, epistemic uncertainty, distrust, and need frustration. Repeated measures require attention to within-person as well as between-person structure; cross-context comparisons require evidence of measurement comparability. Meaning should be measured after exposure and before the next action, need experience at a subsequent brief interval or after an initial attempt, and performance and affect through separate assessments. This ordering reduces retrospective contamination but does not prove causation or exclude rapid reciprocal influences.

Appropriation should be assessed through independently coded behavior before the delayed learning test: whether the learner initiates a criterion, selects relevant evidence, explains an adjustment, requests additional information appropriately, and revises a strategy when evidence changes. Coders should be unaware of assigned condition where feasible, use an explicit rubric, and report agreement. Measures of feedback literacy, metacognitive monitoring, internalization, baseline motor competence, and broader self-regulation provide comparison models. Success at the later transfer task must not itself be scored as appropriation. A cross-validated improvement in prospective prediction would support usefulness; failure to distinguish the process would support using the existing constructs instead.

### 7.2 Manipulate interpretation around identical outputs

Initial experiments should isolate manageable contrasts rather than manipulate a comprehensive ‘supportive AI’ package. A framing experiment changes instructional language while matching output and audience. A visibility experiment changes named disclosure while holding the learner’s reference information and stakes constant. A contestability experiment matches explanation, replay, teacher attention, and nominal choice while varying the right to challenge. A discrepancy study crosses independently established output quality with an opportunity for review. A larger factorial study can subsequently test selected interactions, provided its sample and allocation support those tests. Technical validity should be established before exposure, using task-relevant reference assessment and uncertainty estimates, including checks across body characteristics and movement contexts. Perceived accuracy is measured after exposure and treated as an appraisal, not as proof of technical validity.

For each contrast, preregistration should identify the primary outcome, direction, timing, exclusion rules, manipulation checks, smallest effect of substantive interest, and planned moderators. Checks concern what was changed—perceived framing, audience knowledge, challenge permission, or accessible evidence—rather than successful need satisfaction. The primary analysis should retain participants as assigned; excluding failed manipulation checks can introduce selection bias and belongs, if justified, in sensitivity analysis. Power planning should simulate the intended multilevel model using defensible ranges for effect size, reliability, intraclass correlation, unequal cluster size, and attrition. Interactions generally require separate planning: many repeated trials cannot compensate for too few independently assigned teachers or classes. Imprecise null findings are inconclusive; appropriately powered equivalence or interval-based tests can assess whether a theoretically meaningful effect remains plausible.

Calibration studies should distinguish independently scored cue validity from a displayed confidence label and from the learner’s confidence judgment. Valid and invalid outputs should be balanced or their prevalence explicitly modeled, and the learner’s task knowledge should be assessed so that a missed error is not automatically interpreted as blind trust. Report probability-calibration error, discrimination in reliance, appropriate deferral, and the consequences of following or rejecting each cue. Confidence labels should correspond to actual validation evidence unless an approved, minimal-deception design is indispensable. The ethical safeguards in Section 5.4 apply to all such manipulations.

### 7.3 Observe the process across timescales

Repeated observations should represent feedback events nested within students and classes; teachers require an additional or crossed level when the sampling structure warrants it. Analysis should separate within-student changes from stable between-student differences, account for clustering at the level of randomization, and include time and baseline skill. Prespecified random slopes may be needed where the data support their estimation. Brief temporally ordered measures can relate appraisal at one event to need experience and subsequent regulation, while lagged performance predicts later appraisal as a competing direction. A multilevel association or cross-lagged coefficient alone does not identify a causal mediator. Studies need a stated causal model, assessment of time-varying confounding, and sensitivity analyses rather than treating temporal order as sufficient proof.

Mixed methods are particularly valuable for body–data discrepancy. Stimulated recall can reconstruct what the movement felt like, why an output was accepted or disputed, and how teacher or peer responses changed its meaning. These accounts can refine the construct before large-scale causal claims are attempted. Sensor and performance data can then be triangulated with experience rather than treated as a privileged ground truth in every case.

Longitudinal assessment should distinguish initial exposure, repeated events across a unit, end-of-unit acquisition, delayed retention and transfer, and voluntary activity after the course. Illustrative intervals might include retention after a week, transfer to a changed task at a subsequent session, and activity follow-up several months later; task characteristics and the substantive hypothesis should determine the actual schedule. Repeated follow-ups should measure prior activity, health, habit, identity, access, opportunity, and changes in these conditions. Objective or behavioral activity indicators can complement self-report, with consent and attention to the effects of tracking itself. Attrition, missing events, clustering, and plausible reverse paths must be addressed. This design evaluates Proposition 7 separately from evidence about immediate appraisal or skill transfer.

Covariate selection must follow a stated causal model. Baseline habit or identity may confound a prospective association, whereas changes after instruction could be mediators and should not automatically be controlled away. Public disclosure may also affect classmates assigned to another condition, so researchers should consider class- or session-level assignment and assess interference. These design choices determine the estimand and cannot be replaced by adding all available variables to a regression.

### 7.4 Proposition-to-test map

Table 2 connects each proposition to its primary contrast, candidate measure, assessment time, and analysis. Supplementary Table S2 expands the measurement sequence and validity checks. The model is strengthened by a coherent pattern across these tests, not by counting any favorable motivation, performance, or usage measure as confirmation. The appraisal pathway, added construct labels, and distal participation hypothesis can each fail separately.

**Table 2:** Proposition-to-test map.

Proposition	Primary contrast	Measure and assessment time	Analysis and disconfirming pattern
P1a–c: context	Framing; named disclosure; disagreement × verification. Test each contrast separately.	Informational/controlling appraisal after output; evidence-selection uncertainty before action.	Planned contrasts or interaction in an appropriate multilevel model. Precise nulls after successful manipulation weaken the specified relation.
P2: action	Matched valid discrepancy with versus without an intelligible next-action explanation.	Action comprehension check, then competence satisfaction at a distinct time; record acquisition separately.	Randomized contrast with baseline skill and assignment-level clustering. No meaningful competence difference weakens P2.
P3: contestability	Right to challenge with explanation, replay, teacher contact, and nominal choice matched; cross with output validity.	Permission check; later autonomy satisfaction; trust probability and behavioral reliance on each cue.	Separate autonomy contrast and validity × contestability interaction. Uniform acceptance is not calibration.
P4: disclosure	Same output, norm reference, and grading stakes shown privately or disclosed by name to peers.	Controlling appraisal before subsequent evaluation anxiety; baseline perceived competence.	Disclosure main effect and prespecified competence interaction. Null or reversed specified contrasts weaken P4.
P5: repair	Independently discordant versus concordant output crossed with discrepancy-specific review.	Evidence-selection uncertainty and time to a defensible strategy, before and after review.	Disagreement × review effect on change/duration. No added prediction beyond confusion and uncertainty weakens the added state label.
P6: transfer	Repeated informational versus pressuring arrangement, with technical quality, practice dose, and schedule matched.	Independent monitoring during prompt reduction; later no-AI retention and changed-task transfer.	Randomized total effect; prospective process analysis with stated mediation assumptions. No distinct process or learning effect weakens P6.
P7: participation	Repeated event appraisal across a unit with post-course follow-up.	Autonomous regulation before repeated voluntary-activity measures; baseline and changing maintenance factors.	Multilevel longitudinal model with confounding and attrition checks. Negligible added prospective association weakens this distal pathway.

Measures and schedules are proposed, not validated. Analyses must reflect the assignment and nesting structure; Supplementary Table S2 expands measurement and validity requirements.

## 8 Discussion and implications for teaching

The model proposes an event-level organization of established motivational and feedback mechanisms. Its potential contribution is to distinguish the quality of the AI output from the conditions under which a learner can interpret and use it, and to connect that distinction to independently assessed regulation. Whether this integration explains more than SDT, feedback literacy, or motor-learning accounts considered separately remains open. The following implications are theory-based design principles for testing, not validated prescriptions or estimates of intervention benefit.

One testable principle is to make the purpose, reference, and limits of an output intelligible. Communicating uncertainty and known failure conditions may help learners judge its relevance, but the appropriate detail and timing will depend on task difficulty and learner knowledge. More explanation is not assumed to be universally helpful.

A second principle is to connect a discrepancy to an attainable adjustment. This may improve competence experience beyond providing a precise score alone. It should be tested against equally specific feedback without an action explanation, with practice dose and feedback frequency matched.

A third principle is to limit involuntary evaluative exposure. Private display is a defensible starting arrangement when the pedagogical value of named disclosure has not been demonstrated; voluntary cooperative sharing addresses a different purpose. Decisions about access, retention, and secondary use of movement records also shape what learners expect an output to mean. Consent to participation or recording should not be conflated with consent to public display or later reuse.

A fourth principle is to provide both evidence resources and a credible challenge route. Replay, human explanation, and discrepancy review may be useful for different reasons and should be evaluated separately. A successful repair need not increase agreement with the system: it may justify correcting an output or deferring judgment.

A fifth principle is to examine when prompts can be reduced without losing useful structure. Asking learners to predict, select a cue, and explain their next adjustment may reveal increasing independent regulation. Fading, bandwidth, and learner-controlled schedules should be chosen in relation to skill and task rather than assumed to benefit every learner. Their effects on process measures, acquisition, retention, and transfer require separate tests.

Evaluation should retain the five outcome domains: system engagement, acquisition, retention and transfer, affect and well-being, and continued participation. Lower system use may reflect successful independence, lack of access, or disengagement and is uninterpretable without complementary evidence. Likewise, an immediate gain need not imply sustained well-being or voluntary activity. The model therefore broadens the questions asked of AI-supported instruction while leaving its effectiveness to empirical evaluation.

## 9 Limitations of the theoretical account

The model integrates evidence from PE, motor learning, educational AI, feedback research, and digital behavior-change technology. This breadth is necessary for the problem but creates inferential limits. Findings from written academic feedback, consumer quantification, or fitness trackers cannot be assumed to reproduce in embodied university PE. They support theoretical possibilities and boundary conditions, not direct causal claims about the focal setting.

Blocked translation and self-regulatory appropriation remain provisional process labels. The first may be explained by confusion or epistemic uncertainty, and the second by established feedback-literacy and self-regulation constructs. Conceptual distinctions, candidate indicators, and falsification criteria do not constitute evidence of discriminant validity. Independent studies should permit merger or removal of either label rather than retaining it because it makes the model appear more novel.

The model also foregrounds motivational interpretation without claiming that it is the dominant determinant of performance or participation. Biomechanics, instructional time, task difficulty, health, access, habit, identity, and institutional opportunity can constrain outcomes. Cultural norms may alter the meaning of authority, privacy, bodily display, and peer comparison. The propositions should therefore be tested across contexts rather than assumed universal.

Finally, AI is a moving category. The model does not offer an exhaustive taxonomy of systems, and greater transparency or multimodal interaction may change some proposed risks. Its more durable claim concerns the relation among technical output, pedagogy, social organization, embodied evidence, and learner regulation. New systems can alter that relation, but they cannot eliminate the need to study it.

## 10 Conclusion

AI-generated movement feedback becomes part of an instructional event. Its motivational significance depends on how it is framed, who can judge the learner through it, and what opportunities exist to evaluate its evidential basis. The proposed model applies established motivation and feedback theories to that conjunction in university PE.

The account separates informational and controlling appraisal from subsequent need experience and treats blocked translation as a temporary impediment to evidence-based action. Its principal test is whether these event processes help explain independent monitoring and adjustment and, subsequently, retention and transfer beyond simpler accounts. Continued physical activity remains a secondary, distal hypothesis that requires longitudinal testing under changing opportunities and constraints.

The model’s value will depend on precise contrasts, independent measurement, and willingness to discard unnecessary constructs. Its practical principles should guide comparison and evaluation rather than stand as established recommendations. AI feedback does not motivate by itself; the account specifies how that claim can be investigated without confusing supported performance, learner regulation, and activity beyond the course.

## Data Availability

No datasets were generated or analyzed for this theoretical article. The original theoretical contributions are included in the article and Supplementary material; further inquiries can be directed to the corresponding author.
