# Supplementary material for: AI feedback does not motivate by itself: a motivational-meaning model for university physical education
Source: Front Psychol. 2026 Oct 19;17:1951652. doi: 10.3389/fpsyg.2026.1951652 (PMC13724568; doi:10.3389/fpsyg.2026.1951652)
Supplement: Supplementary file 1 [file Data_Sheet_1.docx]

Supplementary Material

Construct distinctions and a measurement agenda for AI feedback in university physical education.

The following definitions, indicators, and schedules are theoretical proposals. They do not constitute validated measures or report data collected for this article.

# Supplementary Table S1. Proposed construct distinctions and tests of incremental value

The distinctions below are theoretical proposals, not established discriminant validity. Indicators are examples for construct development rather than validated scales. Antecedents are candidate influences and are not diagnostic definitions.

| **Construct** | **Definition** | **Candidate antecedents** | **Independent candidate indicators** | **Distinction and incremental criterion** |
| --- | --- | --- | --- | --- |
| Blocked translation (proposed state) | Inability to establish a sufficiently usable evidential basis for a warranted next action in a feedback episode; it is not a third motivational meaning and may coexist with controlling appraisal. | Conflict between an AI output and another relevant evidence source, coupled with inadequate opportunity to adjudicate the conflict; source authority and opacity are possible moderators. | Pre-review state indicators: reported inability to decide which evidence should guide the next attempt and inability to justify a cue choice, distinguished from productive deliberation. Assess these before observing later action recovery. | Must be distinguishable from confusion, competence frustration, distrust, algorithm aversion, and epistemic uncertainty. Test whether the pre-review state predicts a separately and independently coded post-review action decision or its recovery time beyond these alternatives. Do not reuse the same response or time score as both predictor and outcome. |
| Confusion | Difficulty understanding information or making sense of a situation, without necessarily involving conflicting feedback sources or an inability to choose an action. | Unfamiliar terminology, complex displays, ambiguous instructions, or conflicting information. | Comprehension errors, requests for clarification, and self-reported lack of understanding assessed independently of action selection. | A learner may understand what each source says yet remain unable to adjudicate between them. Blocked translation adds value only if this action-guidance problem is separable from general comprehension difficulty. |
| Competence frustration | The experience of ineffectiveness, failure, or inadequacy in relation to a task; it concerns need experience rather than the evidential basis of a correction. | Repeated failure, unmanageable challenge, or feedback experienced as undermining competence. | Event-specific reports of ineffectiveness or inadequacy using need-frustration measures that do not ask about source disagreement. | Blocked translation can precede a judgment about competence because the learner is uncertain whether the apparent error belongs to the movement, perception, or system. Its measurement and prospective effects must not merely reproduce competence-frustration scores. |
| Distrust | Low confidence in the reliability or credibility of a source, which can be warranted and adaptive. | Documented errors, inconsistent recommendations, prior experience, or low source credibility. | Low assessed probability that an output is valid, selective rejection, or checking behavior compared with an independent validity reference. | A learner can distrust an inaccurate system and still choose a well-supported action from other evidence. Blocked translation specifically concerns inability to establish such a basis, not the absolute level of trust. |
| Algorithm aversion | Reduced willingness to rely on an algorithmic source, considered in relation to available alternatives and the information supplied about their performance. | Observed algorithmic errors, beliefs about algorithmic limitations, or preferences for other decision sources. | Source choice and reliance on algorithmic versus alternative advice under specified and comparable evidence conditions. | Choosing a human source can leave action well guided and is not itself a blocked state. Blocked translation must explain difficulty deciding what to do, beyond source preference or avoidance. |
| Epistemic uncertainty | Uncertainty about the correctness, adequacy, or limits of available knowledge or evidence; it need not prevent warranted action. | Incomplete, noisy, low-confidence, or contradictory evidence and an uncertain task criterion. | Probability judgments, confidence intervals where appropriate, ratings of evidence sufficiency, and sensitivity to acknowledged uncertainty. | The proposed blocked state concerns an action-guidance consequence within an event. Uncertain evidence can still support a prudent next action or help-seeking decision. Blocked translation is redundant if its indicators and consequences add nothing beyond epistemic uncertainty. |
| Self-regulatory appropriation (proposed process) | Acquisition of the capacity to independently perform feedback functions previously supplied by a system: select cues, compare evidence with criteria, choose adjustments, and decide when external help is needed. | Repeated opportunities to interpret, verify, and act on feedback, with suitable guidance and progressively independent judgment. | Quality of cue selection, evidence-based explanations, correction decisions, and justified help seeking when the original system is absent; assessed separately from later motor success. | Must contribute beyond baseline skill, feedback literacy, metacognitive monitoring, autonomous motivation, and broader self-regulation. Regulatory scores should precede and not include the retention/transfer outcomes they are proposed to explain. |
| Feedback literacy | Understandings, capacities, and dispositions for appreciating feedback, making judgments, managing affect, and taking action. | Educational experience, explicit support for evaluating feedback, and opportunities to apply judgment. | Feedback-interpretation and judgment tasks, affect-management and action-use measures, including tasks beyond the focal motor skill. | Feedback literacy can enable appropriation but does not by itself establish that a particular externally supported regulatory function can be executed independently. If literacy measures already capture that transition and predict the same outcomes, appropriation may be a context-specific indicator rather than a distinct construct. |
| Internalization | The process through which externally prompted values or behavioral regulations become more personally endorsed. | Meaningful rationales, need-supportive relationships, and opportunities to integrate the activity with personal goals. | Changes in reasons for acting and the degree of personal endorsement, assessed separately from technical regulatory skill. | A learner may value an activity yet lack the knowledge to monitor movement, or regulate a skill effectively while acting for external reasons. Appropriation concerns learned feedback functions; internalization concerns the regulation and endorsement of action. |
| Metacognitive monitoring | Judgment of one's knowledge, performance, understanding, or uncertainty. | Task experience, explicit prediction and reflection, and exposure to diagnostic evidence. | Accuracy and calibration of performance predictions, error detection, and judgments of uncertainty. | Monitoring is one component of appropriation. The proposed construct additionally requires selecting action cues, coordinating evidence, adapting behavior, and deciding when to seek help without the original system. If these additions are fully captured by established monitoring/control measures, the new label offers limited value. |
| Self-assessment | Evaluation of one's performance or work against explicit or implicit criteria. | Knowledge of criteria, comparison examples, and opportunities to inspect and judge one's performance. | Agreement between self-evaluations and independently assessed criteria, with criterion uncertainty and systematic bias considered. | Accurate self-assessment need not produce an appropriate adjustment or selective help seeking. Appropriation adds value only if its independent action-regulation indicators predict outcomes beyond self-assessment accuracy. |
| Self-regulated learning | A broader process of setting goals, monitoring progress, generating feedback, and adapting strategies and action over time. | Task demands, learner goals and capacities, instructional support, and prior learning experiences. | Goal setting, monitoring, strategy adaptation, help seeking, and recursive regulation across learning episodes. | Appropriation is proposed as a particular transition within self-regulated learning, not a competing general theory. Its usefulness depends on identifying how a system-supported feedback function becomes independently executable and whether that transition improves prediction over suitable self-regulated-learning measures. |

Algorithm aversion is discussed in relation to source selection after observed algorithmic errors (Dietvorst et al., 2015); that literature does not establish the proposed blocked state in PE.

Conceptual anchors: Carless and Boud (2018) for feedback literacy; Butler and Winne (1995) for self-regulated learning; Ryan and Deci (2020) for internalization; Vansteenkiste et al. (2020) for need frustration. Other descriptions are working distinctions for the present comparison, not claims that a separate new scale has been established.

# Supplementary Table S2. Proposed observations, timing, and validity checks

Illustrative time intervals must be justified for the chosen task. All participant research requires applicable prior ethical review and the safeguards described in the main article.

| **Stage / construct** | **Candidate observation** | **Timing** | **Validity and analysis requirement** |
| --- | --- | --- | --- |
| Context and technical validity | Record algorithm/version, task and cue; independently rate output correctness, reference validity, uncertainty, audience, teacher language, assessment and record-access rules. | Before exposure and for each feedback event. | Separate technical validity from perceived accuracy. Assess criterion reliability and relevant body/movement contexts; define matched properties for the planned contrast. |
| Informational / controlling appraisal | Provisional prompts: “What evidence and adjustment options does this message offer?” and “What consequence do you expect if you do not follow it?” | Immediately after exposure, before the next attempt. | Do not use competence, autonomy satisfaction, anxiety, or subsequent success to define appraisal. Test overlap, discriminant validity, and within-person measurement. |
| Blocked translation | Ability to justify which evidence should guide the next action; unresolved choice among cues; time until a defensible action or deferral is selected. | After discrepancy, before action; repeat after review. | Distinguish productive deliberation and justified help-seeking from blockage. Compare confusion, epistemic uncertainty, distrust, and need frustration. |
| Need experience | Event-specific experienced volition, effectiveness, and connection using appropriate need measures. | Baseline; a distinct interval after appraisal; after action where relevant. | Keep item content distinct from message appraisal. Compare changes and reciprocal models; temporal order alone does not establish causation. |
| Trust and reliance | Estimated probability of correctness; accept/check/defer/reject decision; actual following of the cue. | Before receiving outcome information for each scored cue. | Compare judgments with independent validity; report calibration and reliance discrimination separately. Model valid/invalid prevalence and justified deferral. |
| System engagement | Consultations, requests, time with feedback, selective nonuse, and relevant access constraints. | Within event and across the unit. | Use as its own outcome domain. Low use can indicate independence, unavailable access, or disengagement. |
| Acquisition performance | Task-appropriate performance score and practice dose, with feedback conditions recorded. | During supported practice and at unit end. | Use independent/blinded assessment where feasible. Do not condition the primary randomized effect on a post-treatment acquisition value. |
| Appropriation | Initiation of a criterion, cue selection, evidence comparison, explanation of adjustment, and justified help seeking during planned prompt reduction. | Repeatedly across the unit, before delayed learning outcomes. | Use an explicit coding rubric and report agreement. Compare with baseline skill, feedback literacy, metacognition, internalization, and broader self-regulation. Do not include motor success in this process score. |
| Retention / transfer | Persistence of the learned skill and adaptation to a changed task or setting, with original AI support absent or explicitly reduced. | Task-justified delay; e.g., a week for retention and a later changed-task session. | Specify the task and delay in advance; distinguish total intervention effects from assumed mediation. Acquisition-adjusted estimates require a separate justified estimand. |
| Affect / well-being | Event enjoyment or evaluation anxiety; separate measures of well-being across a unit. | After the relevant event; repeated unit-level assessment. | Keep timescales and instruments distinct. Do not infer stable well-being from a single affect item or use affect to define appraisal. |
| Voluntary physical activity | Repeated activity reports plus appropriate behavioral or device measures, distinguishing required from freely chosen participation. | Baseline and prespecified post-course follow-ups, potentially over several months. | Measure health, access, opportunity, habit, identity, and change. Distinguish baseline confounders from post-exposure mediators; assess attrition and residual confounding. |
| Design-wide inference | Manipulation receipt, sample allocation, clustering, adherence, missingness, possible peer spillover, and a specified causal model. | Preregister before recruitment; monitor during collection. | Plan power for primary interactions using the actual multilevel structure. Analyze at the assignment level, separate within- and between-person effects, and use prespecified sensitivity analyses. |

# References

Butler, D. L., and Winne, P. H. (1995). Feedback and self-regulated learning: A theoretical synthesis. Review of Educational Research, 65, 245–281. https://doi.org/10.3102/00346543065003245

Carless, D., and Boud, D. (2018). The development of student feedback literacy: Enabling uptake of feedback. Assessment & Evaluation in Higher Education, 43, 1315–1325. https://doi.org/10.1080/02602938.2018.1463354

Dietvorst, B. J., Simmons, J. P., and Massey, C. (2015). Algorithm aversion: People erroneously avoid algorithms after seeing them err. Journal of Experimental Psychology: General, 144, 114–126. https://doi.org/10.1037/xge0000033

Ryan, R. M., and Deci, E. L. (2020). Intrinsic and extrinsic motivation from a self-determination theory perspective: Definitions, theory, practices, and future directions. Contemporary Educational Psychology, 61, 101860. https://doi.org/10.1016/j.cedpsych.2020.101860

Vansteenkiste, M., Ryan, R. M., and Soenens, B. (2020). Basic psychological need theory: Advancements, critical themes, and future directions. Motivation and Emotion, 44, 1–31. https://doi.org/10.1007/s11031-019-09818-1
